# Effect of Multiple Phosphorus-Nitrogen Flame Retardant on the Properties of PA66

**DOI:** 10.3390/polym17111537

**Published:** 2025-05-31

**Authors:** Haoyang Zhang, Jiyu He, Xiangmei Li

**Affiliations:** 1 School of Materials Science and Engineering, Beijing Institute of Technology, Beijing 100081, China; 3120221107@bit.edu.cn (H.Z.); hejiyu@bit.edu.cn (J.H.); 2 National Engineering Research Center of Flame Retardant Materials, Beijing 100081, China

**Keywords:** PA66, DOPO, flame retardant

## Abstract

PA66 is a widely used engineering plastic, but its flammability reduces safety during application. The 9,10-dihydro-9-oxa-10-phosphaphenanthrene 10-oxide (DOPO) and its derivatives are a class of flame retardants with excellent flame-retardant efficiency, which can significantly improve the flame retardancy of PA66. This work synthesized a DOPO derivative flame retardant, DT, containing multiple P/N elements and comprehensively characterized its structure using FTIR and NMR. Flame-retardant PA66 materials were prepared by twin-screw extrusion blending with PA66, and their thermal stability, crystallization properties, flame retardancy, and mechanical properties were investigated. When the DT content reached 15%, the vertical burning classification test achieved the UL-94 V-0, and the limiting oxygen index (LOI) rose up 27.2%. In the cone calorimeter test, the peak of heat release rate (PHRR) and total heat release (THR) of the material decreased significantly, and a distinct char layer formed, increasing NH_3_ release and decreasing the C-H structure after combustion, improving PA66 flame-retardant properties.

## 1. Introduction

As a common polymer material, PA66 possesses numerous advantages, such as excellent mechanical properties, durable resistance, self-lubrication, chemical resistance, and thermal stability, making it a high-performance material widely used in various fields [1,2,3,4]. However, PA66 is highly flammable, serving as both an ignition source and a path for flame propagation during fires, which significantly contributes to fire initiation and spread. Many applications of PA66 are highly sensitive to fire hazards. Therefore, flame-retardant modification of PA66 is of critical necessity.

Halogen-based flame retardants, recognized as one of the earliest and most efficient flame retardants for PA66 [5], have faced growing restrictions due to the release of hazardous byproducts during their use, posing significant environmental and health risks [6,7,8]. In response, research efforts have transitioned toward environmentally benign phosphorus-nitrogen flame retardants, which offer a greener solution for polymer flame retardancy. Nitrogen and phosphorus are widely recognized as essential flame-retardant elements that play critical roles in fire-resistant applications. By integrating these two elements, researchers can develop advanced nitrogen-phosphorus synergistic flame retardants with enhanced performance [9,10,11]. Phosphorus-based flame retardants operate through dual mechanisms during polymer combustion: in the condensed phase, they decompose to form phosphoric acid that promotes char formation and creates a dense, protective carbon layer to block oxygen diffusion [12], while in the gas phase, they generate PO· radicals that effectively terminate exothermic chain reactions [13,14]. Aluminum diethylphosphinate (ADP) is an exceptionally effective phosphorus-based flame retardant for PA66 [15], demonstrating superior fire-retardant performance through dual-phase mechanisms, with 23.8 wt% phosphorus. Meanwhile, nitrogen-containing flame retardants contribute to fire suppression by releasing inert gases, such as NH_3_ and N_2_ during thermal decomposition [16,17]. Fu et al. [18] synthesized a novel P/N-containing flame retardant, TRFR, and successfully fabricated intrinsically flame-retardant PA66 composites. When incorporating merely 3 wt% TRFR, the modified PA66 exhibited remarkable flame retardancy with a LOI of 29% and achieved a V-0 rating in the UL-94 vertical burning test. Liu et al. [19] synthesized a P/N/Si-containing flame retardant (ZnDSi) using DOPO and prepared flame-retardant PA66 via a blending method. When the ZnDSi content reached 10%, the LOI increased to 29.1%. Compared with pure PA66, the flame-retardant composite exhibited a 35.0% reduction in PHRR and a 28.2% decrease in THR.

In this study, a phosphorus-nitrogen-containing flame retardant, DT, is synthesized from DOPO and subsequently incorporated into PA66 via blending to prepare flame-retardant PA66 composites. The thermal stability, crystallization behavior, and micromorphology of the materials are systematically investigated. The flame-retardant properties are comprehensively characterized through LOI testing, UL-94 rating, and cone calorimetry testing, and the flame-retardant mechanism is discussed.

## 2. Materials and Methods

### 2.1. Materials

PA66 was purchased from Shen Ma Industry Co., Ltd., Pingdingshan, China. DOPO, triallyl cyanurate (TAC), tns-(2.4-di-tert-butyl)-phosphite, antioxidant 1010, and azodiisobutyronitrile (AIBN) were purchased from Macklin Chemical Co. (Shanghai, China). Tetrahydrofuran (THF) and *n*-hexane were obtained from Shanghai Aladdin Bio-Chem Technology Co., Ltd. (Shanghai, China).

### 2.2. Synthesis of DT

**Figure 1:**
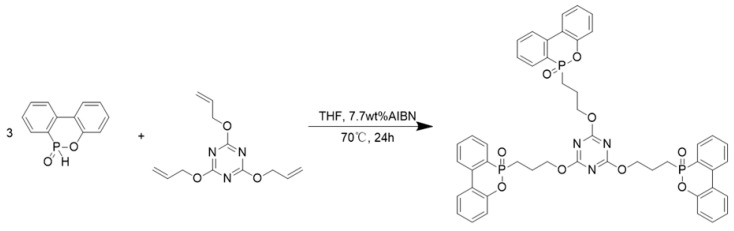
The synthesis method of DT.

The synthesis method of DT is shown in Figure 1. Zang et al. [20] previously synthesized a similar compound through a two-step process: first, the phosphorylation of DOPO with phosphorus oxychloride (POCl_3_), followed by a nucleophilic reaction with trihydroxyethyl isocyanurate. However, this method involves complicated procedures and generates toxic byproducts. In contrast, this study explores a greener and more efficient approach by directly utilizing the addition reaction between DOPO and C=C bonds to synthesize a phosphorus-nitrogen synergistic flame retardant. This method simplifies the synthesis process, avoids hazardous intermediates, and improves overall sustainability. A 250 mL three-necked flask equipped with a condenser and thermometer was charged with 4.32 g of DOPO and 30 mL of THF. The mixture was heated to 70 °C under magnetic stirring until complete dissolution. Separately, 1.25 g of TAC and 0.432 g of AIBN were dissolved in 10 mL of THF and added dropwise into the flask over 1 h using a constant-pressure dropping funnel. The reaction was then allowed to proceed for 24 h. After completion, the THF solution was mixed with 40 mL of hexyl hydride, immediately resulting in precipitation. The solid was collected by filtration and dried in a vacuum oven at 80 °C for 24 h, yielding DT as a pale-yellow powder.

### 2.3. Preparation of Flame-Retardant PA66

Table 1 lists the composition of flame-retardant PA66 materials. PA66 and DT were dried in an oven at 100 °C for 4 h before processing. The uniformly pre-mixed PA66 and DT were processed in a twin-screw extruder to fabricate flame-retardant PA66 composites. Tns-(2.4-di-tert-butyl)-phosphite and antioxidant 1010 were used to avoid decomposition. The temperature of the extruder was maintained at 255–270 °C. The dried pellets of the flame-retardant PA66 materials were molded by the injection molding machine at 275 °C.

### 2.4. Characterization

The nuclear magnetic resonance spectroscopy (^1^H-NMR and ^31^P-NMR) results were obtained with a Bruker Avance III 600 MHz NMR. Samples were implemented with DMSO-d6 as a solvent. An American Nicolet 6700 infrared spectrometer (Waltham, MA, USA) was used to measure the Fourier transform infrared spectroscopy (FTIR) spectra in the range of 400–4000 cm^−1^.

A thermogravimetric analysis (TGA) was performed using a thermogravimetric analyzer STA2500 (Netzsch, Selb, Germany). The samples including DT and flame-retardant PA66 materials were heated from 25 °C to 800 °C under N_2_ conditions at a heating rate of 10 °C/min.

The morphology of flame-retardant PA66 materials and the combustion char residues were analyzed via scanning electron microscopy (SEM; Hitachi Co., Ltd., Tokyo, Japan).

X-ray diffraction (XRD, MiniFlex 600, Rigaku Co., Ltd., Tokyo, Japan) analysis was performed on the flame-retardant PA66 materials at a scanning rate of 2°/minute.

The LOI test was determined on an oxygen index meter (TTech-GBT2406-2, TES Tech Instrument Technologies Co., Ltd., Suzhou, China) with 120 mm × 6.5 mm × 3.0 mm specimens according to ISO 4589-2: 2017.

The UL-94 tests were performed based on a horizontal and vertical burning test instrument (UL94-X, MODIS Combustion Technology Co., Ltd., Qingdao, China) according to the UL 94-2024 testing procedure. The test spline size was 130 mm × 13 mm × 3.2 mm.

The combustion performance was evaluated with a cone calorimeter (FTT-0007, Fire Testing Technology Ltd., East Grinstead, UK) in accordance with the ISO 5660 standard, using specimens with dimensions of 100 mm × 100 mm × 3 mm with 50 kW/m^2^.

The Raman spectra of PA66 and flame-retardant PA66 materials’ residual chars were obtained with a laser Raman spectrometer (HR EV0, Horiba Co., Pairs, France) using a 532 nm helium–neon laser line focused on a micrometer spot on the sample surface.

Tensile strengths were obtained using an Instron 5967 device (Instron, MA, USA). According to the ISO 527-2-2012 test standard, the tensile rate was 20 mm/min. Five specimens were tested for each sample, and the average values were calculated.

## 3. Results

### 3.1. Structural Characterization of DT

**Figure 2:**
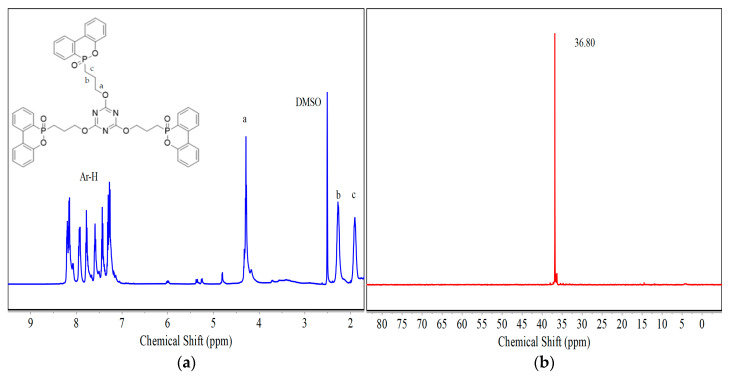
The ^1^H NMR spectra of DT (**a**) and ^31^P NMR spectra of DT (**b**).

NMR and FTIR were used to characterize the structure of the DT. Figure 2 presents the ^1^H and ^31^P NMR spectra of the VDPD flame retardant. In the 1H NMR spectrum of DT, the peaks at 7.24 to 8.22 ppm were assigned to the hydrogen proton of benzene ring, the peak at 4.29 ppm for hydrogen proton of symbol a, the peak at 2.28 ppm for the hydrogen proton of symbol b, and the peak at 1.90 ppm was ascribed to the hydrogen proton of symbol c. In the ^31^P NMR spectrum of the DT flame retardant, the single peak at 36.80 ppm was attributed to the phosphorus atom of CH_2_-P=O, while the characteristic peak of the pure DOPO was not seen. The above analytical results prove that the DT flame retardant has been synthesized successfully.

**Figure 3:**
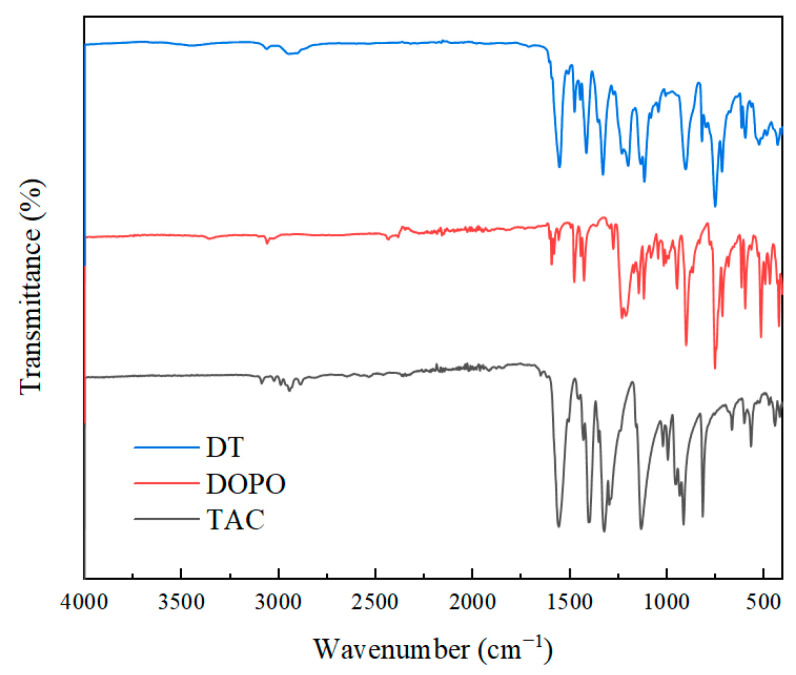
The FTIR spectra of DT, TAC, and DOPO.

Figure 3 illustrates the FTIR spectrum of DOPO, TAC, and DT. The FTIR spectrum of TAC displayed characteristic absorption peaks at 3089 cm^−1^ and 3024 cm^−1^, corresponding to the stretching vibrations of =CH_2_ and =C-H bonds, respectively [21]. In contrast, the spectrum of DOPO revealed distinct peaks at 2434 cm^−1^ (P-H stretching vibration), 947 cm^−1^ (P-H bending vibration), and 1231 cm^−1^ (P=O stretching vibration) [22]. Notably, the FTIR spectrum of the synthesized DT compound showed the complete disappearance of both the P-H bond absorptions from DOPO and the vinyl group peaks from TAC, while simultaneously exhibiting a new P=O stretching vibration at 1200 cm^−1^ that was absent in the original TAC spectrum. These spectroscopic changes provide conclusive evidence for the successful addition reaction between the P-H bond of DOPO and the allyl group of TAC, thereby confirming the formation of the target DT compound through this chemical transformation. 

### 3.2. Thermal Stability

**Figure 4:**
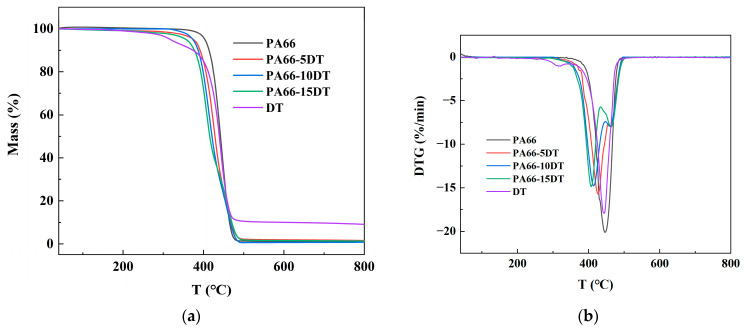
TG (**a**) and DTG (**b**) curves of DT, PA66, and flame-retardant PA66.

The TGA and DTG curves of DT and flame-retardant PA66 are illustrated in Figure 4, and the correlative experiment data are listed in Table 2. The T_5%_ (the temperature corresponding to a 5 wt% weight loss) of pure PA66 was measured at 403.4 °C. With the incorporation of flame-retardant DT, the thermal decomposition of flame-retardant PA66 occurred at lower temperatures, showing a progressive decrease in T_5%_ with increasing DT content. This phenomenon arose because DT decomposes at lower temperatures than the PA66 matrix, causing the flame-retardant components to degrade prior to the PA66 upon heating. The maximum weight loss peak of pure PA66 appeared at 446.1 °C, while the flame-retardant PA66 exhibited two distinct weight loss peaks, with the primary peak shifting to lower temperatures due to the premature decomposition of DT—a trend that became more pronounced with higher DT loading. Additionally, a minor weight loss peak emerged at higher temperatures, corresponding to the thermal decomposition of the remaining PA66 matrix. Although the flame-retardant modification slightly reduced the thermal stability of PA66, it significantly increased the char residue yield, indicating that DT promoted char formation in the PA66 matrix. This enhanced charring effect contributed positively to the flame-retardant performance of the material.

### 3.3. The Dispersion of the Flame Retardants in PA66 Composites

**Figure 5:**
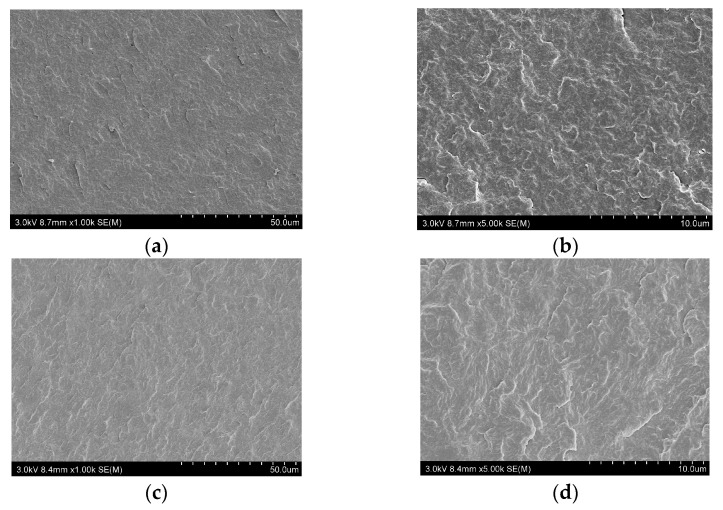
SEM images of the char residues from (**a**,**b**) PA66 and (**c**,**d**) PA66-15DT.

SEM was used to investigate the micromorphology of the brittle failure surfaces of the PA66 composites, as shown in Figure 5. PA66 exhibited a smooth internal surface completely free of impurities, voids, or cracks. Similarly, the fracture surface of PA66-10DT appeared uniform and defect-free, with no observable agglomeration of DT particles, demonstrating excellent dispersion of the flame retardant within the PA66 matrix during melt extrusion processing. This homogeneous distribution minimized the detrimental impact of additive incorporation on the polymer’s intrinsic properties, and significantly enhanced the flame retardant’s effectiveness by ensuring optimal interfacial contact between DT and the PA66 matrix.

### 3.4. Crystallization Properties

**Figure 6:**
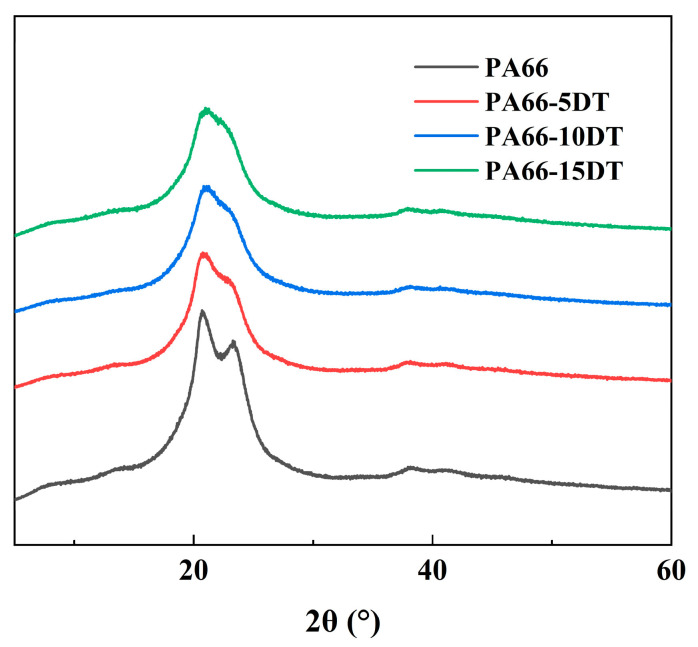
XRD curves of PA66 and flame-retardant PA66 materials.

XRD analysis was conducted on both PA66 and flame-retardant PA66 materials to investigate the effect of DT addition on the crystalline properties, with the XRD curves shown in Figure 6. At room temperature, PA66 typically exhibited α crystal form, characterized by two distinct diffraction peaks at 2θ = 20.70° and 23.28° [23]. Polyamides undergo Brill transition upon heating, a phenomenon first observed in PA66 in 1942 [24]. When heated to approximately 185 °C, PA66 transformed from α crystal form to γ crystal form, which showed a single characteristic peak at 2θ = 21.46°, and reverted to α crystal form upon cooling to room temperature. The XRD curves of flame-retardant PA66 demonstrated that the α crystal form diffraction peaks became less pronounced and tended to merge into a single peak with increasing DT content. This observation indicated that the incorporation of DT promoted the formation of γ crystal form in PA66, and the γ crystal form content further increased with higher DT loading. The phenomenon can be attributed to the hindered rearrangement of molecular chains and hydrogen bonds during cooling after melt extrusion, as the flame-retardant components interfered with the reconstruction of γ crystal form back to α crystal form [25]. More extensive DT addition led to greater disruption of the Brill transition during cooling. Although the flame-retardant PA66 did not completely transform into γ crystal form, the results clearly demonstrated a significant α-to-γ crystal transition, with the γ crystal form proportion increasing progressively with higher DT content.

### 3.5. Flame-Retardant Properties

The results of the LOI and UL-94 of flame-retardant PA66 materials are listed in Table 3. The LOI of PA66 was measured at 22.6%, with severe melt dripping observed during combustion. In the UL-94 vertical burning test, PA66 exhibited pronounced dripping behavior, where the molten droplets ignited the underlying cotton, accompanied by combustion times, t_1_ and t_2_, both exceeding 30 s, resulting in no rating. Upon incorporation of flame-retardant DT, the LOI of PA66 composites progressively increased from 23.4% to 27.2% with higher DT loading. The modified composites formed denser char layers during combustion, effectively suppressing melt dripping. Correspondingly, both t_1_ and t_2_ in vertical burning tests were significantly reduced, showing a dose-dependent improvement trend. PA66-5DT and PA66-10DT samples still produced flaming droplets that ignited the cotton, achieving a V-2 rating. PA66-15DT maintained dripping behavior but the droplets failed to ignite cotton, achieving a V-0 rating.

**Figure 7:**
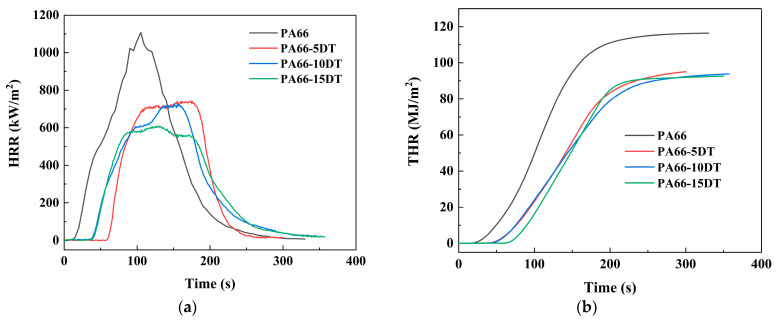
HRR (**a**) and THR (**b**) curves of PA66 and flame-retardant PA66 materials.

The cone calorimetry test serves as a critical method for evaluating the flame retardancy of materials. During testing, the sample was exposed to radiant heat from a conical heater to initiate combustion, while real-time data, including heat release, smoke production, and mass loss, were systematically recorded. This approach provides a comprehensive characterization of material behavior under fire conditions, offering high reliability for flammability assessment. The cone calorimetry test results for PA66 and flame-retardant PA66 are summarized in Figure 7 and Table 4. The cone calorimetry test results revealed that the incorporation of DT significantly enhanced the flame retardancy of PA66. PA66 displayed pHRR and THR values of 1107.7 kW/m^2^ and 116.4 MJ/m^2^, respectively, while the addition of 5 wt% DT reduced these parameters to 741.8 kW/m^2^ (33.0% decrease) and 98.8 MJ/m^2^ (15.1% decrease). This improvement became more pronounced with higher DT loadings, as evidenced by PA66-10DT (728.4 kW/m^2^, 34.2% decrease; 95.8 MJ/m^2^, 17.7% decrease) and PA66-15DT (608.1 kW/m^2^, 45.1% decrease; 94.3 MJ/m^2^, 19.0% decrease). The average effective combustion heat (av-EHC) measurements effectively characterize the burning intensity of gaseous volatiles [26]. During thermal decomposition, PA66 generated combustible gases and molecular fragments that underwent intense oxidation with air, yielding peak av-EHC outputs. For flame-retardant PA66 materials, this value decreased significantly, as phosphorus-containing radicals derived from DT interrupted the combustion chain reaction in the gas phase by scavenging active radicals, ultimately lowering the combustion vigor. The time to ignition (TTI), a critical indicator of material flammability, increased substantially from 12 s for PA66 to longer durations for the flame-retardant PA66, demonstrating that DT addition effectively reduced the material’s ignitability and could provide valuable additional time for escape.

**Figure 8:**
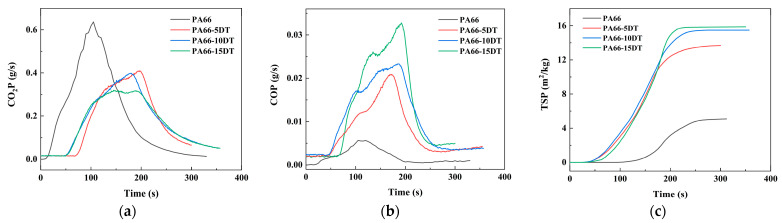
CO_2_P (**a**), COP (**b**), and TSP (**c**) curves of PA66 and flame-retardant PA66 materials.

The carbon monoxide release (COR) and carbon dioxide release (CO_2_R) during combustion effectively characterize material burning behavior, where complete combustion predominantly generates CO_2_, while incomplete combustion produces CO [27]. Compared to PA66, the flame-retardant PA66 materials exhibited significantly increased COR alongside decreased CO_2_R (showed in Figure 8), indicating that the incorporation of DT promoted more incomplete combustion of PA66—a direct manifestation of the flame-retardant effect. This phenomenon became more pronounced with higher DT loadings. Total smoke production (TSP), another critical indicator of combustion completeness [28], showed elevated values in flame-retardant PA66 compared to the pure polymer, further confirming that the additive effectively suppressed complete combustion.

### 3.6. Analysis of the Flame-Retardant Mechanism

**Figure 9:**
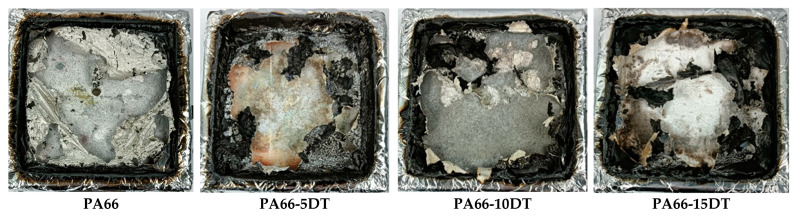
Photographs of residual chars from PA66 samples.

The combustion char layer formation of PA66 materials was thoroughly investigated to analyze the flame-retardant mechanism of DT in the condensed phase. As shown in Figure 9, PA66 left virtually no significant char residue after cone calorimetry testing, with only minimal carbonaceous deposits observed at the mold edges. In contrast, flame-retardant PA66 materials exhibited substantially increased char yields after combustion, clearly demonstrating the flame retardant’s ability to promote char formation.

**Figure 10:**
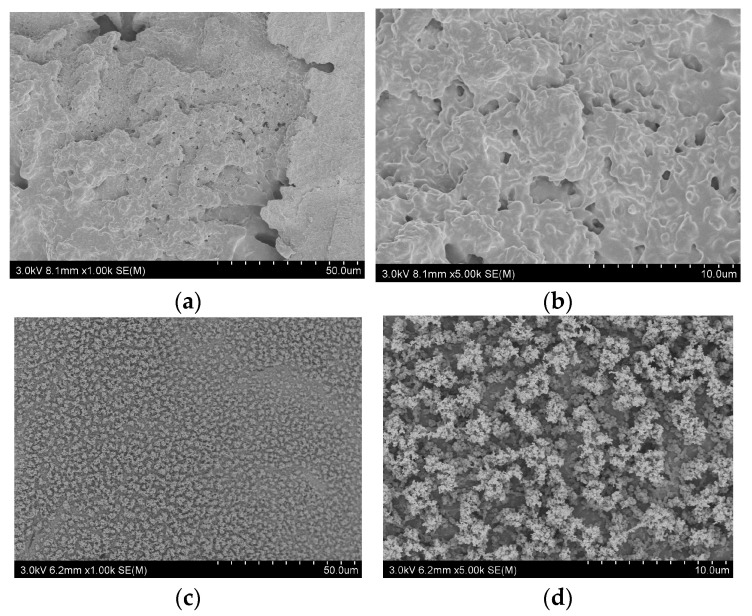
SEM images of the char residues from (**a**,**b**) PA66 and (**c**,**d**) PA66-15DT.

Figure 10 shows the microstructures of the residual chars for pure PA66 and flame-retardant PA66. SEM analysis revealed significant differences in char morphology between PA66 and flame-retardant PA66. The char residue of pure PA66 displayed a highly porous and fragmented structure with numerous cavities and microcracks, resulting in a mechanically weak layer that failed to prevent the escape of flammable volatiles or effectively block heat and oxygen transfer during combustion. In contrast, PA66-15DT formed a continuous, compact char layer with a well-developed dendritic framework structure. This reinforced char architecture exhibited superior mechanical stability and integrity, creating an effective physical barrier that restricted volatile diffusion, hindered heat penetration, and limited oxygen access to the underlying material. 

**Figure 11:**
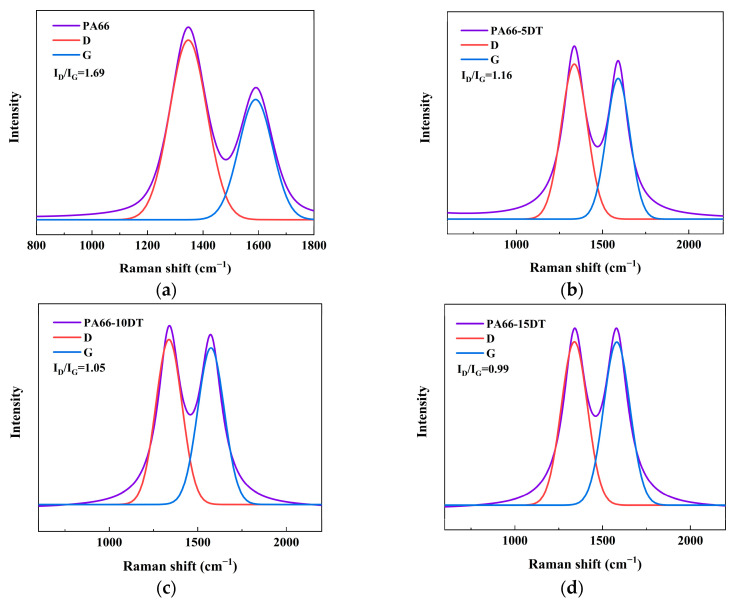
Raman spectra of char residues for PA66 (**a**), PA66-5DT (**b**), PA66-10DT (**c**), and PA66-15DT (**d**). The fitting equation was Gaussian.

Raman analysis of the char residues provided critical insights into the graphitization degree of the carbon layers, where higher graphitization correlates with superior thermal insulation properties [29,30] and enhanced condensed-phase flame retardancy. As shown in Figure 11, the Raman spectra exhibited two characteristic peaks at 1346.5 cm^−1^ (D band, representing disordered carbon) and 1586.2 cm^−1^ (G band, corresponding to graphitic carbon) [31,32], with the intensity ratio (I_D_/I_G_) serving as a quantitative indicator of the graphitization degree. Neat PA66 displayed an I_D_/I_G_ value of 1.69, while the ratio progressively decreased to 1.16 for PA66-5DT, 1.05 for PA66-10DT, and 0.99 for PA66-15DT, demonstrating DT’s ability to promote the formation of more graphitic char layers. This increased graphitization enhanced the char’s barrier properties by improving thermal insulation to reduce heat transfer during combustion [33] and restricting smoke diffusion from the material interior to the environment [34]. The Raman results conclusively established that DT not only increased char yield but also optimized char quality through graphitization enhancement, thereby significantly improving the material’s flame-retardant performance through multiple mechanisms in the condensed phase.

**Figure 12:**
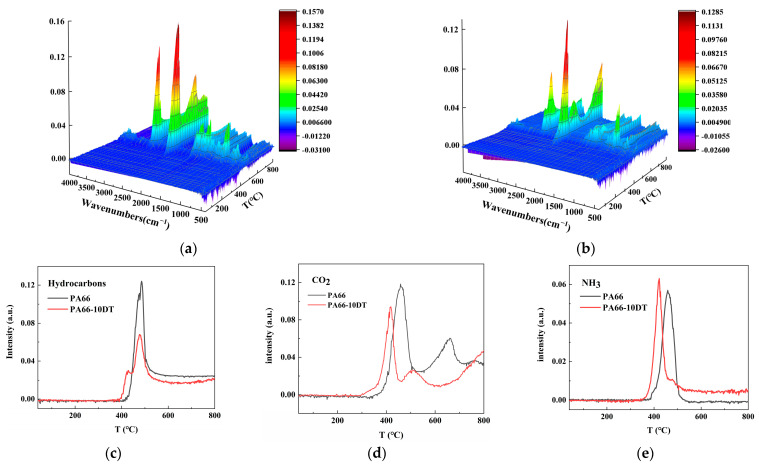
The 3D TG-IR spectra of PA66 (**a**) and PA66-10DT (**b**), release–temperature curves of (**c**) hydrocarbons, (**d**) CO_2_, and (**e**) NH_3_.

TG-IR is used to characterize volatile components during heating in the N_2_ atmosphere. It could help us to investigate the flame-retardance mechanism of DT in the gas phase. As shown in Figure 12, the TG-IR spectra of neat PA66 exhibited two prominent absorption peaks at 2352 cm^−1^ and 2932 cm^−1^, corresponding to the release of CO_2_ and flammable hydrocarbons during thermal decomposition, respectively. Additional characteristic peaks appeared in the ranges of 1827–1624 cm^−1^ (carbonyl compounds) and 977–939 cm^−1^ (NH_3_) [35]. In contrast, the PA66-10DT spectrum demonstrated significantly reduced hydrocarbon emission intensity, with comparative analysis clearly revealing that the incorporation of DT effectively suppressed the release of combustible volatiles during thermal degradation. Additionally, PA66-10DT exhibited reduced CO_2_ emissions, indicating suppressed thermal decomposition, a finding consistent with the cone calorimetry results. What is more, the composite demonstrated increased NH_3_ release compared to pure PA66, which contributed to flame inhibition by diluting the concentration of both flammable volatiles and oxygen in the gas phase, thereby attenuating combustion intensity. This suppression mechanism attenuated the combustion intensity in the gas phase, demonstrating DT’s excellent gas-phase flame-retardant functionality.

### 3.7. Mechanical Properties

**Figure 13:**
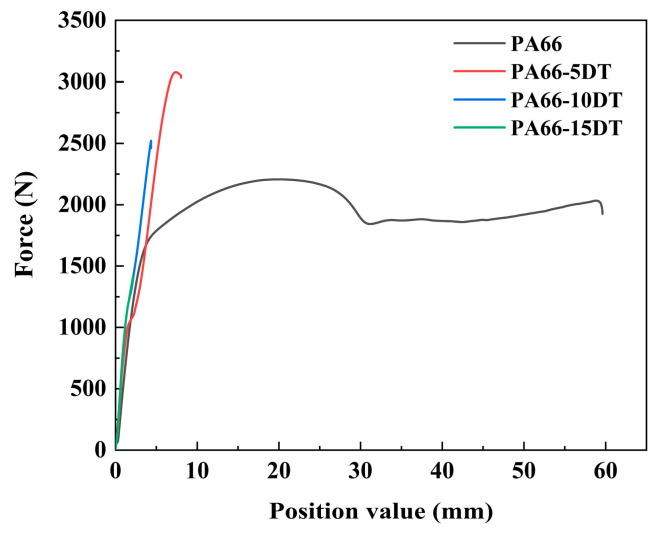
Stretching curves of PA66 and flame-retardant PA66 materials.

Tensile testing was conducted to evaluate the influence of flame-retardant incorporation on the mechanical properties, with results summarized in Table 5. We selected representative samples to plot the stretching curves, as shown in Figure 13. PA66 exhibited typical ductile characteristics during stretching with an elongation at break of 36.88% and tensile strength of 52.99 MPa. However, the addition of DT progressively transformed the material behavior from ductile to brittle, as evidenced by significantly reduced elongation at break with increasing DT content. This mechanical transition originates from crystalline phase modifications—the flame retardant promotes γ-crystal formation, which is more susceptible to structural damage compared to α-crystals [36], leading to easier orientation of crystalline regions under stress and consequent toughness reduction. The excellent compatibility between the flame retardant and PA66 matrix prevented significant stress concentration under load, enabling both PA66-5DT and PA66-10DT to achieve enhanced tensile strengths of 76.9 MPa and 63.0 MPa, respectively. However, when the DT content reached 15%, the material’s tensile strength decreased to 36.2 MPa due to obvious crystalline phase transformation.

## 4. Conclusions

This study successfully synthesized a multiple phosphorus-nitrogen flame retardant, DT, to enhance the flame resistance of PA66. DT was produced through a sustainable synthesis method, attaining a phosphorus content of 10.3 wt%. This efficient methodology demonstrated significant potential for scale-up applications in industrial production.

At 15% DT loading, DT had a good compatibility with PA66, and the PA66 composite achieved a V-0 rating (UL-94) with an LOI of 27.2%, as the molten droplets could no longer ignite cotton. The cone calorimetry test revealed substantial reductions in both the peak heat release rate and total heat release, indicating effective combustion suppression. Microscopic analysis of char showed that the flame-retardant PA66 produced smoother, denser char with fewer surface hole defects compared to neat PA66, Raman spectroscopy confirmed enhanced graphitization, and TGA-FTIR analysis demonstrated DT’s gas-phase activity through reduced hydrocarbon emissions and increased NH_3_ emission during pyrolysis. However, the crystalline phase changes induced mechanical trade-offs, causing the modified PA66 to exhibit brittle fracture behavior.

## Figures and Tables

**Table 1:** Weight ratios of flame-retardant PA66 materials.

Samples	PA66 (g)	DT (g)	Tns-(2.4-di-tert-butyl)-Phosphite	Antioxidant 1010
PA66	1000	0	1	2
PA66-5DT	950	50	1	2
PA66-10DT	900	100	1	2
PA66-15DT	850	150	1	2

**Table 2:** Thermal stability parameters of PA66 and flame-retardant PA66 materials.

Sample	T_5%_ (°C)	T_Max1_ (°C)	T_Max2_ (°C)	Char Residue at 800 °C (%)
DT	317.6	444.6	-	
PA66	403.4	446.1	-	0.79
PA66-5DT	374.8	426.7	461.1	0.89
PA66-10DT	373.4	413.8	459.4	1.32
PA66-15DT	356.2	407.4	462.7	1.56

**Table 3:** LOI and UL-94 data of PA66 and flame-retardant PA66 materials.

Sample	LOI	UL-94				
		t_1_/s	t_2_/s	Dripping	Ignition	Rating
PA66	22.6%	>30	>30	Yes	Yes	NR
PA66-5DT	23.4%	5.7	2.5	Yes	Yes	V-2
PA66-10DT	25.6%	3.8	1.1	Yes	Yes	V-2
PA66-15DT	27.2%	2.3	1.1	Yes	No	V-0

**Table 4:** Cone calorimetry test data of PA66 and flame-retardant PA66 materials.

Sample	pHRR/kW∙m^−2^	THR/MJ∙m^−2^	av-EHC /MJ∙kg^−1^	TTI/s	TSP/m^2^∙kg^−1^	Char Residue/%
PA66	1107.7 ± 9.1	116.4 ± 2.0	32.5 ± 0.8	12 ± 0.3	5.43 ± 0.1	0.4 ± 0.0
PA66-5DT	741.8 ± 5.2	98.8 ± 2.2	29.1 ± 0.7	58 ± 1.2	13.75 ± 0.4	1.3 ± 0.0
PA66-10DT	728.4 ± 2.3	95.8 ± 1.9	28.4 ± 0.4	37 ± 0.9	15.47 ± 0.4	1.7 ± 0.0
PA66-15DT	608.1 ± 4.0	94.3 ± 1.7	26.7 ± 0.4	39 ± 0.7	15.95 ± 0.5	2.1 ± 0.0

**Table 5:** Mechanical properties of PA66 samples.

Sample	Tensile Strength (MPa)	Tensile Modulus (GPa)	Elongation at Break (%)
PA66	53.0 ± 1.0	2.26 ± 0.1	37 ± 1.3
PA66-5DT	76.9 ± 1.7	3.89 ± 0.2	8.8 ± 0.2
PA66-10DT	63.0 ± 1.1	3.49 ± 0.2	2.8 ± 0.0
PA66-15DT	36.2 ± 0.7	2.45 ± 0.1	2.4 ± 0.0

## Data Availability

The original contributions presented in this study are included in the article. Further inquiries can be directed to the corresponding author.
